# Effect of Different Impression and Fabrication Techniques on the Precision of Custom Metal Posts: Fully Digital, Semi‐Digital, and Conventional

**DOI:** 10.1002/cre2.70316

**Published:** 2026-02-19

**Authors:** Masih Rezaee, Mohammadreza Nakhaei, Hamidreza Rajati Haghi, Arsalan Shahri

**Affiliations:** ^1^ Department of Prosthodontics, School of Dentistry Mashhad University of Medical Sciences Mashhad Iran; ^2^ Dental Materials Research Center, Mashhad Dental School Mashhad University of Medical Sciences Mashhad Iran

**Keywords:** 3D printing, CAD/CAM, customized post and core, intraoral scanner, post and core technique

## Abstract

**Objective:**

This study evaluated the effect of different impression and fabrication techniques—conventional casting and 3D printing—on the precision of metal post‐and‐core restorations.

**Methods and Materials:**

A maxillary central incisor was designed in ExoCAD, and STL files of the reference tooth and the “ideal” post were saved; the reference tooth was additively manufactured in metal. Four workflows were compared (*n* = 10/group): (1) conventional impression with a direct resin pattern and casting (CO); (2) semi‐digital impression with an intra‐canal Duralay pattern plus Silicone pick‐up, laboratory scan, and direct metal printing (DS); (3) semi‐digital Full Silicone (putty/wash) impression, laboratory scan, and direct metal printing (FS); and (4) fully digital intraoral scanning with Medit i700 and direct metal printing (FD). For each specimen, the fabricated post STL was compared to the “ideal post” STL in Geomagic Control X to assess precision. Normality was tested with Shapiro–Wilk, and between‐group comparisons used one‐way analysis of variance in SPSS (*α* = 0.05).

**Results:**

In forty specimens (FD, DS, FS, CO; *n* = 10/group), three outcomes were analyzed: In‐Tol, Over‐Tol, and Under‐Tol. In‐Tol differed significantly among groups (*p* < 0.001); Tamhane's T2 indicated FD > DS, FS, CO (*p* = 0.006, < 0.001, < 0.001). Over‐Tol differed significantly (*p* < 0.001); FD < FS (*p* < 0.001), FD < CO (*p* = 0.021), and DS < FS (*p* < 0.001). Under‐Tol also differed significantly (*p* < 0.001); DS > FD (*p* = 0.027), DS > FS (*p* = 0.011), and CO > FS (*p* = 0.016).

**Conclusion:**

Fully digital, directly printed Co–Cr posts exhibited superior precision compared with conventional and semi‐digital workflows. While conventional casting remains reliable, direct metal printing appears to be a practical and potentially easier alternative.

## 1 Introduction

Restoring severely damaged teeth, especially in the esthetic zone, is challenging due to limited coronal structure and high cosmetic demands. Endodontic posts and cores—made from zirconia, fiber composites, or custom cast alloys—are commonly used to provide retention and support for crowns (Schwartz and Robbins 2004; Carvalho et al. 2018; Çelik Öge et al. 2024).

Endodontic posts can be prefabricated or customized. Prefabricated posts are convenient but may not fit the canal well, increasing dislodgement risk, whereas customized cast posts offer better canal adaptation, torsional resistance, and preservation of tooth structure (Heydecke and Peters 2002; Morgano and Milot 1993; Morgano et al. 2004; Sarkis‐Onofre et al. 2014; Fuss et al. 2001). Despite concerns about metal posts causing root fractures (Assif and Gorfil 1994), recent evidence shows no significant difference compared to fiber posts (Figueiredo et al. 2015). A precise, well‐fitted post with a uniform cement layer is essential for long‐term success (Schmage et al. 2005).

Conventional customized posts and cores can be fabricated using direct or indirect techniques. The direct method allows precise intraoral adjustments but is time‐consuming and highly technique‐dependent, while the indirect method is faster and suitable for multiple teeth but relies on laboratory accuracy and may introduce impression errors (Rayyan et al. 2016; Sabbak 2002; Ravanshad and Ghoreeshi 2003; Al‐Omari and Zagibeh 2010). Although both methods are considered gold standards with comparable fit and retention, they are technique‐sensitive, often require multiple appointments, and lack reproducibility, with potential discrepancies that can compromise retention or cause root fracture (Goodacre et al. 2003; Moshonov et al. 2005; Fasbinder 2010; Pitigoi‐Aron et al. 2012; Guachetá et al. 2022).

Digital workflows offer effective alternatives to conventional methods by streamlining procedures, improving efficiency, and reducing chairside time, costs, and patient discomfort. Using CAD/CAM technology through milling or three‐dimensional (3D) printing, digital techniques enhance consistency, reproducibility, and quality in post and core fabrication. However, despite their growing popularity, the accuracy of CAD/CAM restorations compared with traditional lost wax techniques remains uncertain (Lee and Gallucci 2013; Yuzbasioglu et al. 2014; El Kerdani and Roushdy 2017; Papadiochou and Pissiotis 2018; Daher et al. 2024).

3D printing has transformed dental practice by enabling the production of various appliances and restorations using polymer and metal materials (Dawood et al. 2015; Tahayeri et al. 2018; Tian et al. 2021). Polymers are widely used for their ease of fabrication and high accuracy (Henprasert et al. 2020), though they often remain partially polymerized after printing and therefore require post‐curing, a process that can cause shrinkage and compromise fit (Bagheri and Jin 2019). Castable wax resins address this limitation by eliminating post‐curing, reducing shrinkage, and improving the accuracy of restorations (Guachetá et al. 2022).

The precision of metal post‐and‐core restorations is essential for their long‐term clinical success; however, limited evidence exists regarding the influence of impression and fabrication techniques on their accuracy. As metal 3D printing gains clinical relevance, this study aimed to compare the 3D geometric precision (surface accuracy) of the 3D geometric precision of custom metal posts fabricated by four workflows: (1) conventional direct resin‐pattern casting; (2) semi‐digital resin pattern with silicone pickup followed by direct metal 3D printing; (3) semi‐digital full elastomeric impression followed by direct metal 3D printing; and (4) fully digital intraoral scanning followed by direct metal 3D printing. The null hypothesis was that there would be no significant difference in precision between metal post‐and‐core restorations fabricated using conventional and digital/semi‐digital impression and manufacturing techniques.

## 2 Methods

### 2.1 Study Design

This was an in‐vitro comparative accuracy study, conducted at the Department of Prosthodontics (Mashhad University of Medical Sciences, Iran) in 2025. The local Ethical Committee of Mashhad University of Medical Sciences reviewed and approved this in vitro study with the protocol number of IR.MUMS.DENTISTRY.REC.1404.058, on October 18, 2025.

### 2.2 Sample Size

The sample size was based on the between‐group difference in adaptation reported by Piangsuk et al. (2023) (mean difference ≈ 1.07, SD ≈ 0.48 [units consistent with the primary adaptation metric]). Using the two‐means formula:

n=((Z₁₋α⁄₂+Z₁₋β)²)/(((μ₁−μ₂)/s)²),

with *α* = 0.05 (two‐sided; *Z* = 1.96) and power = 0.80, yielded *N* = 5 per group. To enhance precision and allow for potential exclusions, the target was increased to 10 specimens per group (total *N* = 40).

### 2.3 Reference Model (Master Tooth) Design and Fabrication

**Figure 1:**
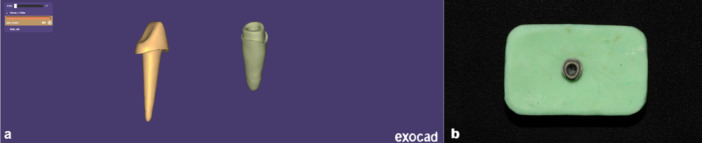
Reference tooth and ideal post designed (a); subsequently, the tooth was metal 3D‐printed and finally mounted (b).

A maxillary central incisor with normal root/crown anatomy and a butt‐joint shoulder finish line was designed for a single‐crown preparation in ExoCAD (3.2 2024 Elefsina; Exocad GmbH) with an intracanal length of 10 mm, an orifice diameter of 3.5 mm, and a 1‐mm post‐stop margin thickness. A corresponding “ideal” custom post‐and‐core perfectly matching the root canal was also designed. Both the tooth STL and the “ideal post” STL were exported and archived for subsequent alignment and analysis (Figure 1a).

To enable repeated impressions without material degradation, the CAD tooth was additively 3D‐printed in metal (SLM, Alloy Co‐Cr); Riton T150, Guangzhou Riton Additive Technology Co. Ltd., China). This metal master tooth was mounted and used for all impression and scanning procedures in all groups (Figure 1b).

### 2.4 Experimental Groups (*N* = 10 Specimens Per Group; Total *N* = 40)

**Figure 2:**
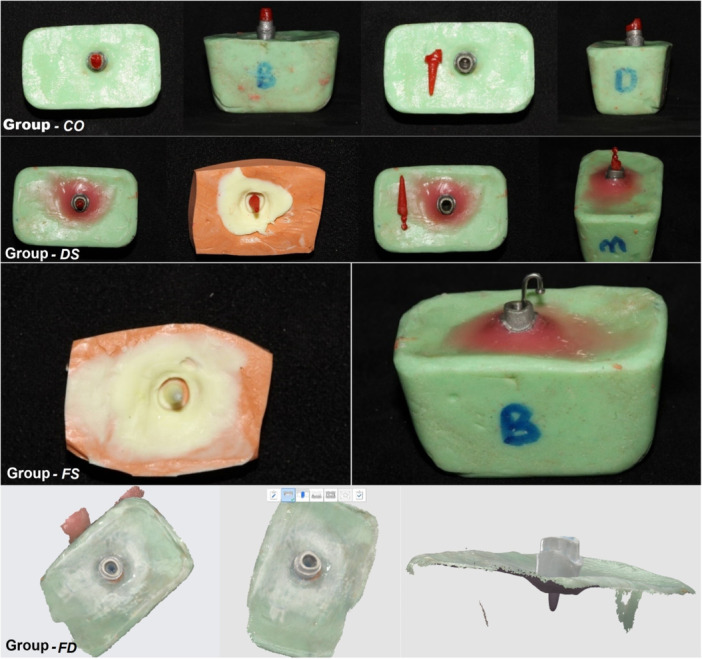
Representative steps of the four fabrication workflows.

For each workflow, 10 independent specimens were fabricated from the metal master. The four groups are presented in Figure 2 and are defined as follows:

- G1 — Conventional (CO):

A direct intra‐canal pattern was made with resin pattern (Duralay, Reliance, USA) on the metal master. The coronal margin was finalized on the master tooth with a dental spatula. The resin pattern margins were polished on the external surface. Patterns were sprued, and placed in a casting ring, invested with a phosphate‐bonded investment (Cera‐Fina; Whip Mix), and cast with a cobalt‐based dental alloy (Co–Cr alloy, Magnum Fulgens; Mesa). Castings were divested and finished; the internal surface was air‐abraded with 150 μm Al₂O₃ at 0.5 MPa before evaluation to remove residual investment from the post.

- G2 — Semi‐digital (DS) ‐ (Duralay + silicone pickup → lab scan → CAD → direct metal 3D print):

An indirect record of the canal was first obtained by making a Duralay resin post pattern intra‐canally on the master; the pattern was picked up in a silicone impression (PVS, putty, BONASCAN; DMP, Greece). The impression (with the captured canal) was lab‐scanned (Shining 3D DS EX Pro H, Hangzhou, China). A post‐and‐core was designed in ExoCAD (3.2 2024 Elefsina; CAD Software; Exocad GmbH), then directly 3D‐printed in metal (SLM, Alloy Co‐Cr (N03); Riton T150, Guangzhou Riton Additive Technology Co. Ltd., China). Cement gap was considered 30 µm in the ExoCAD software for all metal 3D printed posts.

- G3 — Semi‐digital (FS) ‐ (full elastomeric impression → lab scan → CAD → direct metal 3D print):

A full silicone putty‐wash impression of the master was made (PVS; putty + light‐body, BONASCAN; DMP, Greece). During the wash phase, a rounded‐tip endodontic needle (26 gauge) was placed into the canal to support wash flow and ensure full capture of the canal geometry. The final impression was lab‐scanned, the post‐and‐core was CAD‐designed in ExoCAD, and a direct metal 3D‐printed post was fabricated as in G2.

- G4 — Full‐digital (FD) ‐ (intraoral scan → CAD → direct metal 3D print):

The metal master tooth (including its intracanal area) was scanned using an intraoral scanner (Medit i700, Medit, Seoul, Korea; IOS software version: 2.4.6). The post‐and‐core was designed in ExoCAD and direct metal 3D‐printed as in G2/G3.

All fabricated posts were placed on silicone putty and scanned using a Shining Pro laboratory scanner. The STL files were then exported and archived for subsequent alignment and analysis.

### 2.5 Primary Outcome Measurements— 3D Geometric Precision

Accuracy was defined as the surface deviation between each fabricated post STL and the CAD “ideal post” STL. All analyses were performed in Geomagic Control X (version 2022; 3DSystems, USA).

Alignment protocol was accomplished by using best fit alignment and then 3D Compare to measure the In‐Tolerance volume (%), Over‐Tol volume (%), Under‐Tol volume (%). All recorded variables are defined as follows:

- In‐Tol (%) — percentage of post surface points falling within the tolerance band (± T). Higher values indicate better precision.

- Over‐Tol (%) — percentage of points above +T (positive deviations).
- Under‐Tol (%) — percentage of points below −T (negative deviations).

**Figure 3:**
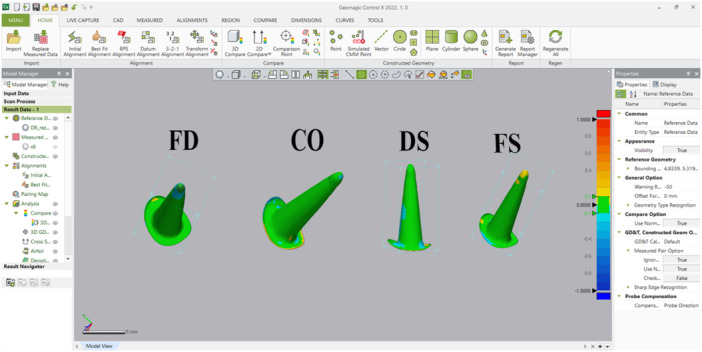
Geomagic Control X 3D Compare environment used for surface‐deviation analysis. The color bar (mm) encodes deviations: green = within tolerance (In‐Tol), red = positive deviation (Over‐Tol), blue = negative deviation (Under‐Tol).

**Figure 4:**
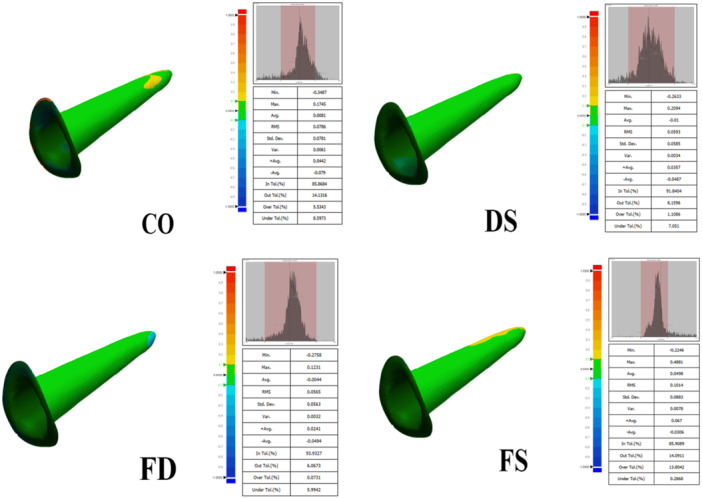
Geomagic Control X 3D Compare analysis results for each sample.

Notes: Geomagic 3D Compare outputs deviations also generates color deviation maps (green = in‐tolerance, red = over‐tolerance, blue = under‐tolerance), as illustrated in Figure 3. Additional variables can be measured from the 3D Compare results table; however, only these three variables were used, as they are more clinically relevant for posts and cores and directly related to the objectives of our study (Figure 4).

The In‐Tol threshold for Surface Comparison was set at 50 µm, consistent with the CAD cement gap used for digitally produced zirconia and metal posts and cores (Perlea et al. 2024). Points within this range were considered accurately fitting, allowing quantification of pre‐cementation surface precision.

All post STL files (fabricated posts and corresponding CAD “ideal post” files) were trimmed from the occlusal seat margin upward. Finally, the match between each fabricated post STL file and the corresponding CAD “ideal post” STL file was evaluated using these three variables to represent the accuracy of each fabrication technique.

Moreover, two metal posts were printed from the ideal post STL, then scanned and compared with the “ideal post” STL to verify the precision of the metal printer and the validity of the procedure.

### 2.6 Procedure Validity, Randomization, and Blinding

Reference (CAD control) showed almost complete scanning and printing accuracy with the following parameters: In‐Tol = 100%, Over‐Tol = 0%, Under‐Tol = 0%. No randomization was needed as all groups used similar master tooth. Moreover, all ExoCAD designing, and Geomagic measurements was conducted by 1 dental laboratory technician who was blinded to group allocation.

### 2.7 Statistical Analysis

Outcomes (In‐Tol, Over‐Tol, and Under‐Tol) were summarized as mean ± SD; group means with 95% CIs were graphed. Normality was assessed with the Shapiro–Wilk test (All variables showed normal distribution); one‐way analysis of variance (factor: Group, 4 levels) was applied to each outcome. When variances were unequal, pairwise comparisons used Tamhane's T2. The α level was 0.05. Analyses were performed in SPSS (version 22.0, SPSS Inc., IBM, Armonk, New York, USA).

## 3 Results

**Figure 5:**
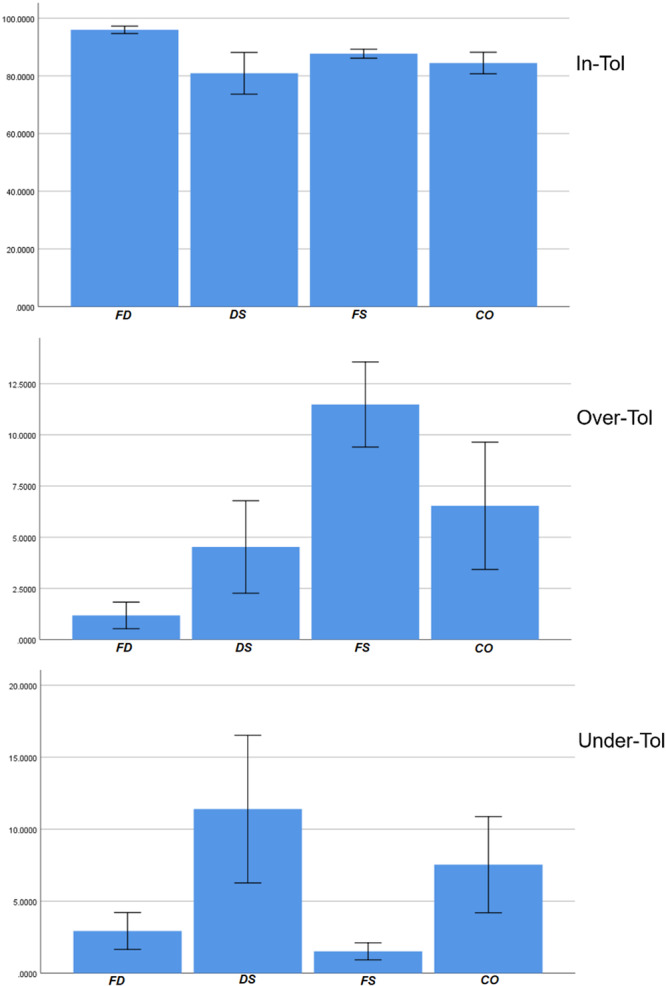
Mean and 95% confidence intervals of the three variables by group.

Forty specimens were analyzed (FD, DS, FS, CO; *n* = 10/group). Three outcomes were evaluated (In‐Tolerance, Over‐Tolerance, and Under‐Tolerance). Normality was supported by the Shapiro–Wilk test. All data are presented at Table 1 and Figure 5.

**Table 1:** Between‐group comparison in all outcomes.

Outcome	Group*	*N*	(Minimum)–(Maximum)	Mean (SD)	One‐way ANOVA
In‐Tolerance	FD^**a**^	10	(94)–(100)	95.94 ± (1.81)	*F* = 12.04 *p* < 0.001
	DS^**b**^	10	(64)–(94)	80.88 (10.10)	
	FS^**b**^	10	(85)–(92)	87.66 (2.17)	
	CO^**b**^	10	(75)–91)	84.43 (5.24)	
Over‐Tolerance	FD^**a**^	10	(0)–(2)	1.18 (0.91)	*F* = 19.50 *p* < 0.001
	DS^**ab**^	10	(0)–(10)	4.52 (3.16)	
	FS^**c**^	10	(7)–(14)	11.49 (2.91)	
	CO^**bc**^	10	(1)–(15)	6.53 (4.35)	
Under‐Tolerance	FD^**ab**^	10	(0)–(6)	2.93 (1.79)	*F* = 10.54 *p* < 0.001
	DS^**c**^	10	(0)–(21)	11.39 (7.17)	
	FS^**a**^	10	(0)–(3)	1.52 (0.83)	
	CO^**bc**^	10	(1)–(14)	7.53 (4.67)	

Abbreviation: SD, standard deviation.

* According to Tamhane post hoc test, different lowercase letters indicate a significant difference between the two different groups in each variable analysis.

The lowest and highest means for In‐Tol occurred in DS and FD. One‐way ANOVA indicated between‐group differences (*p* < 0.001). Tamhane's T2 post hoc showed FD > DS, FS, CO (*p* = 0.006, < 0.001, < 0.001). Other pairwise comparisons were not significant.

The lowest and highest means for Over‐Tol occurred in DS and FS. One‐way ANOVA indicated between‐group differences (*p* < 0.001). Tamhane's T2 showed FD < FS (*p* < 0.001), FD < CO (*p* = 0.021), and DS < FS (*p* < 0.001). Other pairwise comparisons were not significant.

The lowest and highest means for Under‐Tol occurred in FS and DS. One‐way ANOVA indicated between‐group differences (*p* < 0.001). Tamhane's T2 showed DS > FD (*p* = 0.027), DS > FS (*p* = 0.011), and CO > FS (*p* = 0.016). Other pairwise comparisons were not significant.

## 4 Discussion

A well‐fitting post minimizes cement film thickness and intra‐tooth stresses (Schmage et al. 2005). This in vitro study evaluated how different impression and fabrication techniques—conventional and digital—affect the precision of the final metal post to the tooth. Four custom metal post‐and‐core workflows were compared, focusing on the impression method and the laboratory fabrication pathway: (1) conventional with a direct resin pattern and casting (CO); (2) semi‐digital with an intra‐canal resin pattern plus silicone pickup, extraoral scanning, and direct metal printing (DS); (3) semi‐digital with a full silicone impression, extraoral scanning, and direct metal printing (FS); and (4) fully digital with intraoral scanning and direct metal printing (FD).

For surface precision (In‐Tol), the FD group showed the highest precision, whereas DS, FS, and CO did not differ significantly from one another. For Over‐Tol, group FS exhibited higher positive deviations than group DS. For Under‐Tol, group CO showed more negative deviations than group FS, and group DS also showed more negative deviations than group FS.

Group FD demonstrated the greatest percentage of surfaces within tolerance (In‐Tol) and the lowest positive/negative deviations. In a comparable study, Hendi et al. (2019) reported that outcomes improve as workflows move toward either end of the spectrum (fully conventional or fully digital), which aligns with the present finding for In‐Tol in group FD. Mechanistically, the fully digital route (FD) involves the fewest cross‐overs between conventional and digital steps, thereby reducing cumulative error potential. Also our results are consistent with the findings of Jensen and Sayardoust (2024) who reported that fully digital SLM fabrication of custom‐made posts and cores provided a superior internal fit compared to conventional methods. This supports our observation of higher precision in the fully digital group.

In this study, an innovative approach was employed to quantify pre‐cementation precision using surface‐based 3D comparison in Geomagic Control X, reporting In‐Tol, Over‐Tol, and Under‐Tol. This approach identifies bulging/nodular or recessed areas on the 3D model, though it does not measure the actual cement thickness after cementation. Supporting this software's use, Piangsuk et al. (2023) aligned resin patterns and metal posts (pre‐ and post‐fabrication) in Geomagic and used surface deviations and color maps to assess the dimensional stability of direct, milled, and printed workflows; Abad‐Coronel et al. (2023) likewise imported paired STLs, performed initial alignment and best‐fit, and reported deviations within a ± 0.1 mm tolerance band—together indicating that non‐cemented, contact‐free assessment can be performed with good precision and repeatability. By contrast, many studies such as Kanduti et al. (2021) and Liu et al. (2019) used micro‐CT to quantify volume/area of the cement space across levels; Jensen and Sayardoust (2024) used the silicone replica technique to assess internal fit at seven defined points, and measurements were taken with a microscope. Each method has distinct strengths and limitations; a combined surface‐ and volume‐based assessment is recommended for comprehensive evaluation.

Pairwise comparisons revealed no significant differences among DS, FS, and CO, consistent with Alnazzawi et al. (2024), who reported no significant dimensional accuracy differences between printed and cast metal posts and noted the time and cost advantages of digital workflows. Similarly, Piangsuk et al. (2023) found that, after final adjustments, the accuracy of direct, milled, and printed posts did not differ significantly, with printed resin patterns showing better dimensional stability than milled patterns, and digitally produced metal posts achieving accuracy comparable to directly fabricated ones.

To interpret deviation trends, group FD, which showed the smallest deviations, can be set aside. For Over‐Tol, the largest positive deviations occurred in the FS group (full silicone impression), likely due to elastomeric dimensional changes and the longer manipulation time required for wash placement with a lentulo, producing slightly oversized posts. For Under‐Tol, groups using Duralay resin in impression steps (DS and CO) exhibited greater negative deviations, attributable to acrylic shrinkage and surface bubbles, which increase cement‐gap areas. Non‐uniform Duralay surfaces may also affect surface‐based precision analysis in Geomagic Control X, and lab scanning of resin (DS) versus higher‐fidelity BONASCAN material (FS) likely contributed to observed differences.

Beyond precision, clinical performance also depends on retention and fracture strength. Hendi et al. (2019) reported that conventional cast posts had higher pull‐out forces and smaller apical gaps than digital posts, though differences remained clinically acceptable. Alqarni et al. (2022) found that printed or milled titanium posts exhibited greater tensile forces than cast base‐metal and zirconia posts, with zirconia showing the lowest retention. Regarding fracture strength, milled Co–Cr posts demonstrated the highest values, while SLM posts (as used here) performed similarly to cast posts, with negligible effects from print orientation (Bilgin et al. 2016; Kalyoncuoğlu et al. 2023). Thus, selecting a fabrication route should consider surface precision, retention, strength, and time/cost together.

Multiple materials can serve as post substrates (Jung et al. 2007); in this study, Co–Cr was used for all printed groups, indicating that observed differences were driven mainly by impression and fabrication methods rather than alloy. Other studies highlight material effects: Alzaid et al. (2024) found cast‐gold produced a thinner, more uniform cement film than printed/milled titanium, while Alqarni et al. (2022) reported higher retention for printed/milled titanium than base‐metal or zirconia, with zirconia lowest. Digital workflows also offer practical access to materials like titanium (Liao et al. 2023), which can be challenging to cast traditionally. Material selection should therefore balance mechanical, biological, and esthetic considerations.

The accuracy of intraoral scanning for post spaces is influenced by canal depth, diameter, and scanner type, with narrower or deeper canals generally increasing deviations (Alqahtani et al. 2024). Laboratory studies using a 10‐mm conical cavity reported mean scanner errors of 10–30 µm; for example, the Medit i700 (without adjacent teeth) produced 16.4, 12.4, and 9.8 µm at 0–3, 3–6, and 6–9 mm, respectively, while some devices could not fully capture deeper areas and adjacent teeth reduced model quality (Dupagne et al. 2023). Comparative data among Trios, iTero, and Medit devices indicate inter‐scanner differences, and decreasing cervical diameter (e.g., 2.5 mm vs. 3 mm) can increase deviations, highlighting the importance of scanner choice and multi‐angle acquisition protocols (Meshni et al. 2024). In our study, a single, idealized canal geometry was used, which allowed controlled comparisons but limits generalizability. The relatively wide canal orifice (3.5 mm) and absence of adjacent teeth likely facilitated scanning of apical regions, yet future studies incorporating variable canal geometries are needed to fully assess metal‐print post accuracy in clinical scenarios.

Digital workflows for custom metal posts can achieve high structural precision while reducing fabrication time. Clinically, this is advantageous for severely compromised teeth and canals with thin dentin walls, where a precise fit and uniform, thin cement layer is critical. By eliminating many error‐prone laboratory steps, tooth preparation is minimized, and faster, even single‐visit delivery becomes feasible with the appropriate equipment. Additionally, storing STL files and design parameters allows for easy remanufacture in case of failure or loss. Optimized digital workflows can thus enhance efficiency, accuracy, and speed simultaneously (Al‐Rubaye and Elsubeihi 2024; Abdulkarim et al. 2024; Mahato et al. 2024; Bernauer et al. 2023).

The reference tooth was printed in metal to accommodate the Duralay‐containing groups; printing it in resin could have caused chemical bonding with Duralay or required substantial spacers, both of which would compromise accuracy. A single metal master with constant anatomy was used, which does not reflect the diversity of natural canal morphologies. While surface‐based Geomagic analysis identifies areas prone to microleakage and surface mismatch, it does not directly measure retention or total cement thickness. Moreover, only one intraoral scanner and one metal 3D printer were evaluated, making the findings system‐specific. Future studies should (i) print as many master teeth as test posts to enable both computational and retention testing—with a metal master STL allowing re‐printing if failure occurs during pull‐out tests; and (ii) assess different master designs, as the single‐unit design here lacked undercuts, which could influence the performance of methods like FS.

## 5 Conclusion

A fully digital workflow (intraoral scanning + direct metal printing) achieved the highest pre‐cementation precision (In‐Tol) for Co–Cr posts, outperforming conventional and semi‐digital methods. The three non‐fully‐digital workflows (CO, DS, FS) showed no significant differences in In‐Tol. Deviation patterns were technique‐dependent: resin‐based steps (DS, CO) exhibited more under‐tolerance (larger cement gaps), whereas full PVS (FS) showed more over‐tolerance (oversized posts), providing guidance for laboratory adjustments. These results indicate that, under laboratory conditions, scan‐to‐print can achieve surface precision comparable to or exceeding lost‐wax methods, while conventional casting remains reliable. However, since only pre‐cementation geometric precision was evaluated, conclusions regarding clinical performance—such as retention, fracture resistance, and behavior in varied canal anatomies—should be drawn cautiously.

## Author Contributions

Mohammadreza Nakhaei and Hamidreza Rajati Haghi conceptualized and designed the study. Masih Rezaee and Arsalan Shahri performed the experimental procedures. Arsalan Shahri conducted the statistical analysis. Arsalan Shahri and Masih Rezaee drafted the manuscript, and all authors revised and approved the final version.

## Ethics Statement

All procedures were approved by the ethical committee of Mashhad University of Medical Sciences, Iran (IR.MUMS.DENTISTRY.REC.1404.058).

## Conflicts of Interest

The authors declare no conflicts of interest.

## Data Availability

The data of the current study are available from the corresponding author on reasonable request.
